# Health Services for Buruli Ulcer Control: Lessons from a Field Study in Ghana

**DOI:** 10.1371/journal.pntd.0001187

**Published:** 2011-06-21

**Authors:** Mercy M. Ackumey, Cynthia Kwakye-Maclean, Edwin O. Ampadu, Don de Savigny, Mitchell G. Weiss

**Affiliations:** 1 School of Public Health, College of Health Sciences, University of Ghana, Accra, Ghana; 2 Swiss Tropical and Public Health Institute, Basel, Switzerland; 3 University of Basel, Basel, Switzerland; 4 Ga-West Municipal Health Administration, Amasaman, Ghana; 5 National Buruli Ulcer Control Programme, Accra, Ghana; Ghana Health Service, Ghana

## Abstract

**Background:**

Buruli ulcer (BU), caused by *Mycobacterium ulcerans* infection, is a debilitating disease of the skin and underlying tissue. The first phase of a BU prevention and treatment programme (BUPaT) was initiated from 2005–2008, in the Ga-West and Ga-South municipalities in Ghana to increase access to BU treatment and to improve early case detection and case management. This paper assesses achievements of the BUPaT programme and lessons learnt. It also considers the impact of the programme on broader interests of the health system.

**Methods:**

A mixed-methods approach included patients' records review, review of programme reports, a stakeholder forum, key informant interviews, focus group discussions, clinic visits and observations.

**Principal Findings:**

Extensive collaboration existed across all levels, (national, municipality, and community), thus strengthening the health system. The programme enhanced capacities of all stakeholders in various aspects of health services delivery and demonstrated the importance of health education and community-based surveillance to create awareness and encourage early treatment. A patient database was also created using recommended World Health Organisation (WHO) forms which showed that 297 patients were treated from 2005–2008. The proportion of patients requiring only antibiotic treatment, introduced in the course of the programme, was highest in the last year (35.4% in the first, 23.5% in the second and 42.5% in the third year). Early antibiotic treatment prevented recurrences which was consistent with programme aims.

**Conclusions:**

To improve early case management of BU, strengthening existing clinics to increase access to antibiotic therapy is critical. Intensifying health education and surveillance would ultimately increase early reporting and treatment for all cases. Further research is needed to explain the role of environmental factors for BU contagion. Programme strategies reported in our study: collaboration among stakeholders, health education, community surveillance and regular antibiotic treatment can be adopted for any BU-endemic area in Ghana.

## Author Summary

Buruli ulcer (BU), caused by *Mycobacterium ulcerans* infection, is a debilitating disease of the skin and underlying tissue which starts as a painless nodule, oedema or plaque and could develop into painful and massive ulcers if left untreated. Using a combination of quantitative and qualitative methods, the study assessed the effectiveness of the BUPaT programme to improve early detection and management of BU in an endemic area in Ghana. The results of the study showed extensive collaboration across all levels, (national, municipality and community), which contributed to strengthening the programme. Health staff were trained to manage all BU cases. School teachers, municipal environmental staff and community surveillance volunteers were trained to give the right health messages, screen for detection of early cases and refer for medical treatment. WHO-recommended antibiotics improved treatment and cure, particularly for early lesions, and prevented recurrences. Improving access to antibiotic treatment is critical for early case management. Health education is required to emphasise the effectiveness of treatment with antibiotics to reduce deformities and the importance of seeking medical treatment for all skin lesions. Further research is needed to explain the role of environmental factors in BU contagion.

## Introduction

In the absence of a proven strategy for preventing infection, control of Buruli Ulcer (BU) relies on efficient health services to prevent progression of pre-ulcerative conditions and treat ulcers. According to the World Health Organisation (WHO), service delivery is the primary function of any health system and entails the provision of “effective, safe, good quality care to those that need it with minimal waste”, [1] and to address health care needs through promotion, prevention, treatment and rehabilitation. WHO defines a health system as “all organisations, people and actions whose primary intent is to promote or to restore health” [1].

Buruli ulcer, caused by *Mycobacterium ulcerans* infection is a debilitating disease of the skin and underlying tissue which starts as a painless nodule, oedema or plaque and could develop into painful and massive ulcers if left untreated [2]. It is the third most common mycobacterial pathogen of humans, after *M. tuberculosis* (tuberculosis) *and M. leprae* (leprosy), but the most poorly understood [2], [3] . Even though case fatality is low, morbidity is high for all age groups [3]–[5] and the socio-economic implications to the individual and cost of management to the health system are enormous [6], [7].

Surprisingly, estimates of Disability Adjusted Life Years (DALYs) for Buruli ulcer, like other neglected tropical diseases (NTDS) such as guinea worm, endemic syphilis and food-borne trematode infections are not explicitly stated [8]. BU has been reported in more than 33 tropical and sub-tropical climates particularly West African countries [2], [9], and Ghana reports an average of 1000 cases each year [9]. The first case of BU was reported in Ghana in 1972 in the Ga-district. [10]. A national case search in 1998 indicated a national prevalence of 20.7/100,000 and a prevalence of 87.7/100,000 for the former Ga-district (now the Ga-West and Ga-South municipalities), the fifth most endemic in the country, yet with the highest burden in terms of healed and active lesions [11].

The first phase of a BU prevention and treatment programme (BUPaT) was initiated from 2005–2008, in the Ga-West and Ga-South municipalities in the Greater-Accra region, Ghana, to increase access to BU treatment and improve early case detection. Before the inception of the BUPaT programme, surgery was the main treatment for all BU patients. There was limited accessibility to treatment since all surgeries had to be done at the Amasaman hospital (AH), the main treatment and referral hospital for all BU cases in the Ga-West municipality. Antibiotic treatment had not been introduced and health staff had limited expertise in surgical procedures and BU case management.

The BUPaT programme employed WHO-recommended strategies which are: Building capacity of nurses and other para-medical staff for effective case detection, and management at designated health centres; training of community-based surveillance volunteers (CBSVs), school teachers, other health workers and traditional healers (THs), to enhance BU knowledge for early detection; establishing a community-based surveillance system with the help of CBSVs; compiling a database; providing surgical and antibiotic therapy for all BU patients [12].

These strategies were undertaken by a health team that consisted of staff of the national Buruli ulcer control programme (NBUCP), the BUPaT programme from World Vision Ghana, the health directorates of the Ga-West and Ga-South municipalities, surgeons from the Korle-Bu teaching hospital in Accra, the municipal school health education programme (SHEP) coordinator, municipal environmental health officers (MEHOs), CBSVs, THs and community members. This paper assesses achievements of the BUPaT programme and lessons learnt for early case detection, case management and access to treatment in a BU-endemic rural area of Accra. It also considers the impact of the programme on broader interests of the health system.

## Methods

### Study setting

This study was conducted from November 2008 to July 2009 in the Ga-West and Ga-South municipalities. The Ga-West municipality shares boundaries with the Ga-South municipality to the west. It has a population of 215,824 inhabitants of which 48.2% are males and 51.8% are females. About 60% of the municipality's landscape is rural with about 200 scattered communities; 40% is urban and peri-urban and is densely populated. The population of the Ga-South municipality is estimated at 210,727 distributed in 362 communities. Like the Ga-West municipality, 48.2 % inhabitants are males and 51.8% are females. The population is mainly concentrated along the peri-urban areas of the municipality.

At the time of conducting the survey, the Ga-West and Ga-South municipalities were known as the Ga-West District. The Ga-West district covered the same geographical area as these two municipalities (Ga-West and Ga-South). Through a government legislative instrument, the Ga-West district was divided into two separate municipalities in 2009 for easy governance and accessibility of health services.

Since 1999, BU continues to be a major cause of morbidity in both municipalities with increasing numbers of related disabilities. Health services are provided by 3 main government health centres, Weija hospital, Amasaman hospital (AH) and the Obom Health Centre (OHC), a few private clinics, and family planning and maternity homes.

### Study design

The study employed a mixed methods approach using quantitative and qualitative methods to assess the effectiveness of the BUPaT programme in improving early detection and management of BU in the Ga-West and Ga-South municipalities. This approach provided the needed framework for obtaining, understanding, comparing and cross-validating contextual information from providers and beneficiaries of BU-related health service delivery strategies. The various methods were complementary; emerging and divergent issues arising during the course of one approach were clarified with another. Aside document reviews which was ongoing over the course of the study, all the other approaches followed sequentially.

### Stakeholder forum (SF)

A day's forum was held with thirty five (35) persons that included the programme manager of the NBUCP, the municipal chief executive (MCE) of the Ga-West municipality, some municipal health staff, officials and BUPaT staff of World Vision Ghana, doctors and nurses from the AH and the OHC, officials from the Ghana education service, CBSVs and MEHOs. This forum reviewed the BUPaT programme activities, explored issues regarding health services delivery, capacity of health staff to deliver BU-related services and the integration of programme activities in communities and schools. Health service delivery interventions such as the role of CBSVs in case detection, early reporting and strengthening existing clinics in the community to increase access to health services were discussed. Consideration was given to community participation, sustainability of the programme as well as the next steps for future strategies at BU control.

### Documents review

Quarterly and annual BUPaT programme reports were studied to provide background information and insights into programme objectives, strategies and challenges.

### Key informant interviews (KIIs)

KIIs were held with the municipal health director (MHD) of the Ga-West municipality, the programme managers of the NBUCP and the World Vision Ghana, Ga-West municipality development programme. These persons were selected because of their pivotal role in the BUPaT programme. KIIs highlighted issues on access to care, successes and challenges of the programme and emphasised strategies requiring further strengthening.

### Patients' records review

Already analysed records of 297 patients from the AH were reviewed to indicate the statistical trend, demographic characteristics of patients, assess indicators of treatment procedures, effectiveness of treatment and outcomes.

### On-site clinic visits

Visits were made to the OHC and the Kojo Ashong clinic to assess the effectiveness of decentralising treatment and management of Buruli ulcer.

### Focus group discussions (FGDs)

One FGD each was held in three randomly selected endemic communities (Kojo Ashong, Avornyokope, and Balagono). Each focus group was made up of 10 purposively selected persons, comprising treated and discharged adults, and care-takers of child patients. FGDs examined community perceptions about the programme, school-based strategies, and the effectiveness of medical treatment, particularly antibiotic treatment. FGDs also considered challenges and concerns that were raised at the SF and KIIs regarding low hospital/clinic attendance and late reporting.

### Analysis of data

Information from BUPaT programme reports were subjected to a thematic content analysis. Themes were derived from activities that formed health service delivery strategies. Thematic related activities, (community-based surveillance, community education, school-based education and antibiotic therapy) were examined for their contribution to awareness creation, access to timely treatment, care and management of BU, and how best they addressed the overall aim of the BUPaT programme. Consideration was also given to the extent of collaboration and coordination of activities among stakeholders. Documented successes and challenges of the programme as well as those mentioned at the SF and during KIIs and FGDs were noted.

Discussions and interviews from the SF and the KIIs were subjected to a thematic content analysis. Interviews were conducted in English and tape-recorded. During the interviews, elaborate notes were taken and themes that emerged during these discussions were noted. Subsequently, interviews were transcribed using Microsoft Word. Transcriptions were translated and edited, preserving the original style and context. The authors developed a coding framework based on themes pertinent to the main features and strategies of the BUPaT programme [13]. These themes included ‘collaboration’, ‘health services’, ‘health education’, ‘access and utilisation’, ‘coverage’, ‘adequacy of facilities’, ‘antibiotics’, ‘surgery’, ‘complications’, ‘recurrence’, ‘patients’, ‘feeding’, ‘transportation’, ‘community’, ‘traditional healers’ and ‘community-based surveillance volunteers’.

FGDs were conducted and recorded electronically in the local languages. Notes on content and context referred to recurring themes. FGDs were translated into English and transcribed using Microsoft Office Word. Similar to the procedure for analysing the SF and the KIIs, transcriptions were subjected to a thematic content analysis. A coding scheme was devised using themes that clarified perceptions of health service delivery strategies and medical treatment. These themes included ‘volunteers’, ‘treatment’, ‘late treatment’, ‘traditional healers’, ‘herbal treatment’, ‘medicines’ and ‘costs’.

Observations during clinical visits were recorded in a notebook. We paid attention to the type of treatment given to patients, number of patients who received antibiotic care and documentation of patient data. Subsequently, clinical registers were examined to ascertain the extent to which patients adhered to treatment.

Patient data captured on the WHO BU01 forms had already been extracted and analysed by health staff and therefore there was no need for any further analysis.

### Ethics Statement

The study was approved by the ethical review committee of the Ministry of Health, Ghana, and the ethics commission of Basel (Ethikkommission beider Basel (EKBB)) in Switzerland. Verbal consent was preferred to written ones since it did not pose any psychological threat and reassured all interviewees of anonymity. Both ethical review boards approved of verbal consent as long as participation in the study was voluntary, participants had been informed of the study aims and had the opportunity to ask questions. Prior to the start of all interviews, interviewees were informed about: the study aims, their rights to withdraw participation from the study, the intended use of findings to improve BU related health services and, for publications in academic journals and reports. Informed verbal consent was witnessed by two members of the BUPaT team who were not members of the research team.

## Results

### Collaboration and networking of all partners and stakeholders

Programme documents indicated that the BUPaT programme was initiated by WVG and the MHD of the Ga-West Municipality. The Municipal Chief Executive (MCE) of the Ga-West Municipality and the NBUCP were engaged at the design stage. At the onset, a Memorandum of Understanding (MOU) was formalised with the MCE to ensure partnership with the local government authorities, and subsequently the municipal health staff and beneficiary communities. Table 1 shows a timeline of BU activities in the country and study municipalities.

**Table 1:** Timeline of Buruli ulcer programmes and activities in Ghana.

Dates and References	
1971 [4]	First case of BU identified in a patient from the Ga district
1989 [11]	96 cases of Buruli ulcer infection were discovered in the Asante Akim North District in the Ashanti Region of Ghana.
1993[18]	A passive surveillance system for reporting Buruli ulcer was initiated in Ghana by the Ministry of Health.
July 1998[26]	Signing of the Yamoussoukro declaration on Buruli ulcer in Yamoussoukro, La Côte d'Ivoire, by the Director-General of the WHO and Heads of State of Ghana, Benin and Côte d'Ivoire. These governments agreed to mobilise resources to establish national Buruli ulcer control programmes, conduct epidemiological surveys on BU and establish surveillance systems with technical support from the WHO.
June-July 1999 [19]	The Ghana Ministry of Health conducted a national case search on BU in the entire country. A total of 5,619 persons were identified with BU lesions at various stages in all 10 regions of the country. The national prevalence rate was computed as 20.7/100,000 and the Ga-district prevalence rate was 87.7/100,000 for active lesions.
2002 [27]	The establishment of the Ghana National Buruli ulcer Control Programme in accordance with the Yamoussoukro declaration.
July – August 2005[21]	Community-based study on knowledge, attitude and practice of Buruli ulcer conducted in the Ga-West district of Ghana.
2005	Buruli ulcer Prevention and Treatment Programme commenced in the Ga-West and Ga-South municipalities of the Greater Accra region of Ghana.
March 2009[28]	Cotonou declaration adopted in Cotonou, Benin, by the WHO Director-General, Minister of health, Ghana, other West African presidents and participants, to take all the necessary measures to alleviate the suffering caused by Buruli ulcer, and to contribute to further enhancement of knowledge about the disease.

Programme documents, the SF and KIIs indicated a strong partnership with the NBUCP which provided technical expertise and training of health staff. To create awareness and ensure the participation of civil society, programme documents revealed that the BUPaT programme was duly launched at a durbar in the capital of the municipality, Amasaman. THs, WVG staff, officials from the NBUCP, municipal executives, health staff, teachers, CBSVs, school children and community members were in attendance.

Programme documents indicated that the core management team of the programme was the WVG Ga-West municipality manager, the MHD and the Municipal SHEP Coordinator. Selection of members for this team was guided by the main activities of the programme which were community and school health education, screening, medical treatment, surgery and wound care, community surveillance, documentation and compilation of a patients' database. Some individuals from the Municipal Health Management Team (MHMT) served as focal persons for various aspects of the programme. WVG too had a focal person for the programme, officially known as the BUPaT programme coordinator. This person was responsible for financial issues, logistics, monitoring, collation and analysis of patients' records, and served as a liaison between WVG and the MHMT. The MHD and the MHMT coordinated health activities related to BU.

A coalition of stakeholders including health, environmental, educational professionals, CBSVs and traditional rulers was formed to ensure diversity of expertise as well as community participation. As a practice, stakeholder meetings were organised quarterly to report on the progress of the programme. Additionally, a monitoring team comprising selected individuals from the stakeholder group was constituted to evaluate programme goals and objectives and follow-up on treated and discharged patients.

### Training of health staff and other stakeholders for increased awareness, case detection, community-based surveillance and case management

According to programme documents, 120 CBSVs, 40 THs, 4 MEHOs and 113 teachers from 60 schools were trained to detect early cases of BU in communities and refer promptly to health facilities for treatment. BU information was included in the school curriculum. Documents and narratives from the SF revealed that officials from the NBUCP also trained 40 nurses in BU case-detection, surveillance, wound care and prevention of disabilities associated with BU. After training, these nurses were distributed among the municipal health facilities: AH, OHC and two newly opened health centres (one each at Dome Sampahman and Kojo Ashong communities). Programme documents, the SF and KIIs also revealed that refresher courses were held quarterly for nurses, CBSVs and MEHOs. The NBUCP arranged for two surgeons from the Korle-Bu teaching hospital to perform weekly surgical operations on patients.

### Health education, screening and community-surveillance to improve early detection and treatment of cases

Programme documents indicated that the BUPaT programme aimed to reduce BU-related suffering and disability through early detection and treatment of pre-ulcer cases. The programme therefore employed health education to create awareness, screening and surveillance to detect all forms of BU, particularly early cases to increase early reporting for medical care, antibiotic care, wound dressing and surgery.

According to programme documents, AH staff and the SHEP coordinator conducted BU education and screening in 80 schools. Health staff, BUPaT programme staff and CBSVs combined efforts to conduct health education in over 600 communities. Sometimes these education campaigns culminated in BU screening. MEHOs also organised night-time film shows on BU and followed up the next day for screening. CBSVs mounted intense surveillance in their localities and paid random home visits to screen and verify suspected cases of *M. ulcerans* infection.

### Improved clinical treatment and case management of BU

Programme documents, the SF and KIIs revealed that the WHO-recommended antimicrobial (rifampicin and streptomycin) therapy was introduced at the beginning of the BUPaT programme in 2005, and administered to all patients. Health staff were trained in the appropriate protocols to be observed when administering these antibiotics. By policy, BU treatment is covered under the National Health Insurance Scheme (NHIS). Narratives from the SF and the KIIs indicated that these antibiotics which are anti-tuberculosis drugs were provided by the NBUCP. Medicines and dressings were provided by the Ministry of Health through the NBUCP and sometimes by World Vision Ghana when stocks were exhausted. The SF forum also mentioned that surgery was carried out at least once a week at the AH by a surgical team from the Korle-Bu teaching hospital. Documents highlighted the infrastructural limitations of the OHC and the Kojo Ashong clinic that made it impossible for surgical operations to be carried out there.

At the Kojo Ashong clinic, located 20 kilometres from the AH, in an endemic community, BU care was limited to antibiotic therapy. At the time of the research team's visit, 4 patients had been registered: 2 female adults and 2 male children. During the visit, the team observed treatment of the children and 1 adult. The children proceeded to school after treatment. In addition to antibiotic care, the OHC performs minor excisions; patients requiring major surgery are referred to the AH. At the time of the team's visit, 9 persons (6 children and 3 adults) had already received treatment, though clinic records indicated that 24 patients (15 children and 9 adults) had been registered. Patient records also showed that only those 9 registered patients had regular treatment and they lived close to the OHC. Although rehabilitation of patients with disabilities is an integral component of BU care, all key informants admitted that this did not feature on the programme's agenda for lack of capacity and infrastructure. One key informant explained:

*We are exploring the possibility of referring patients who need to be rehabilitated but who will pay for this service?*

### Compilation of a patient database

The NBUCP trained all health staff on the appropriate use of the stipulated WHO BU01 forms to record patient information, disease outcomes, and clinical and surgical procedures. Analysed data from these forms indicate that 297 patients were treated from June 2005 to June 2008. Children below 15 years constituted nearly half (146; 49%) of all admissions over the 3-year period. Patients presenting with ulcers formed the majority of all clinical forms: 52 (52.5%) in the first, 62 (73%) in the second and 67 (59.3 %) in the third yearly periods. There were 14 (14%) patients with recurring lesions (June 2005-May 2006) and none during the latter yearly periods (Table 2).

**Table 2:** Patient characteristics and clinical forms of Buruli ulcer (2005–2008).

	Yearly periods *		
	2005–2006 (%)	2006–2007(%)	2007–2008 (%)
	N = 99	N = 85	N = 113
**Patient characteristics**			
**Age**			
Less than 15years	56 (56.6)	40 (47.1)	50 (44.2)
15–49	38 (38.4)	38 (44.7)	52 (46.0)
Above 49 years	5 (5.1)	7 (8.2)	11 (9.7)
**Sex**			
Male	41 (41.4)	41 (48.2)	62 (54.9)
Female	58 (58.6)	44 (51.8)	51 (45.1)
**Clinical form**			
Nodule	22 (22.2)	3 (3.5)	18 (16.0)
Plaque	22 (22.2)	11 (13.0)	10 (8.8)
Oedema	2 (2.0)	7 (8.1)	7 (6.2)
Ulcer	52 (52.5)	62 (73.0)	67 (59.3)
Mixed	1 (1.0)	2 (2.4)	10 (8.8)
Osteomyelitis	0 (0.0)	0 (0.0)	1 (0.9)
**Patient classification**			
New	85 (86.0)	85 (100.0)	113 (100.0)
Recurrent	14 (14)	0 (0.0)	0 (0.0)
**Specimen taken for lab confirmation**			
Yes	15 (15.2)	19 (22.4)	28 (24.8)
No	84 (84.8)	66 (77.6)	85 (75.2)

**Source:** Patient data 2005–2008, Amasaman hospital.

*Since the BUPaT programme was initiated in June 2005, a yearly period was calculated from June to May the next year.

Except for the last yearly period (June 2007-May 2008) where only 34.5 % of patients healed without deformities, more than 60 percent of patients healed without deformities for the first and second years (Table 3). The proportion of patients that reported early and therefore were given only antibiotic treatment over the programme period was encouraging, 35.4% in the first yearly period, 23.5% in the second yearly period and 42.5%, in the third. The programme recorded 4 BU-related deaths (Table 3). Utilisation of services for BU increased over the three-year period. Of the 297 BU patients treated during this period, 113 were treated in year 3 (38.0%) compared with 85 (28.6%) in year 2, and 99 (33.3%) in year 1 (Table 3). Irrespective of these achievements, a significant proportion of patients either absconded treatment, or were lost to follow-up (14.1% in the first yearly period 9.4% in the second yearly period and 14.2%, in the third) (Table 3).

**Table 3:** Treatment types, outcomes and surgical procedures for Buruli ulcer patients (2005–2008).

	Yearly periods *		
	2005–2006 (%)	2006–2007 (%)	2007–2008 N (%)
	N = 99	N = 85	N = 113
**Disability present on admission**			
Limitation present	14 (14.0)	19 (22.4)	32 (28.3)
No limitation present	85 (86.0)	66 (77.6)	81 (71.7)
**Treatment types**			
Surgery only	37 (37.4)	4 (4.7)	0 (0.0)
Antibiotics only	35 (35.4)	20 (23.5)	48 (42.5)
Antibiotics and surgery	27 (27.3)	61 (71.8)	65 (57.5)
** **Surgical procedures**			
Excision only	24 (37.5)	16 (24.6)	33 (50.8)
Skin grafting	36 (56.3)	41 (63.1)	28 (43.1)
Amputation	1 (1.6 )	2 (3.1)	2 (3.1)
Wound debridement	3 (4.7)	6 (9.2)	2 (3.1)
**Treatment outcomes**			
Healed without deformity	67 (67.7)	53 (62.4)	39 (34.5)
Referral	13 (13.1)	14 (16.5)	6 (5.3)
Healed with deformity	4 (4.0)	9 (10.6)	14 (12.4)
Absconded / lost to follow-up	14 (14.1)	8 (9.4)	16 (14.2)
Died, Buruli ulcer related	1 (1.0)	1 (1.2)	2 (1.8)
Still on admission	0 (0.0)	0 (0.0)	36 (31.9)

**Source:** Patient data 2005–2008, Amasaman hospital.

*Since the BUPaT programme was initiated in June 2005, a yearly period was calculated from June to May the next year.

**Surgical procedures explains treatment types for patients that had ‘surgery only’ and ‘antibiotics and surgery’.

### Improving access to treatment by providing incentives to surgeons; feeding and transport to patients

WVG provided cash incentives to plastic surgeons to ensure continuity of surgical operations. It was apparent from programme documents that the BUPaT programme supported in-patients and in some cases relations or caregivers with two meals (breakfast and lunch). Other organisations and individuals within and outside the municipalities also contributed towards feeding of patients either through cash donations or food items. All transport costs of patients and accompanying CBSVs to the AH, OHC, and patients who were referred to Korle-Bu hospital for specialised care were reimbursed. Key informants remarked that although feeding and refund of transport costs was not considered in the original programme design, it had to be incorporated later taking into consideration the poverty of programme beneficiaries, and remarked that good nutrition enhanced the healing of wounds.

All 3 key informants and stakeholders highlighted the high costs of treatment which placed a huge strain on the limited health budgets of the municipalities. They perceived a major difficulty in sustaining the programme if World Vision Ghana withdrew its financial support especially in the absence of government budgetary funding.

### Achievements of the BUPaT programme

Among the contributions of the BUPaT programme to BU control, the following achievements are notable: improved collaboration among stakeholders, early case detection and treatment, increased community awareness of the priority of BU and improved access to treatment. Promoting awareness and access to improved services has made it possible to minimise surgical interventions, which the earlier programme had relied on almost exclusively.

The priority of early detection and treatment highlighted in programme documents (quarterly and annual reports), was consistent with accounts in the SF, KIIs and FGDs. FGD participants commended the community and school health education programmes, use of media especially documentary films and the efforts of the CBSVs. Participants regarded these strategies as helpful for increasing their awareness, promoting disease surveillance and encouraging early presentation of affected persons for treatment. A participant at the SF summarised the achievements of the programme as follows:

*The success of this programme is due to the extensive collaboration and networking of all those involved across all levels; national, municipality and community. Community-based surveillance volunteers are our foot soldiers in the community and they have done extremely well in surveillance, case detection and referral. They are the link between the communities and the municipal hospital.*

Our three key informants asserted the primary success of the BUPaT programme in managing BU was best indicated by the increasing number of patients receiving treatment at the AH over the course of the programme period. Statistics from the Ga-West municipality showed that prior to establishing the programme there were 70 cases in 2001, 82 in 2002, 83 in 2003 and 71 in 2004 [14]. In 2005, when the BUPaT programme commenced, AH recorded 99 cases and the number increased to 113 in 2008 over the 3-year period of the programme.

**Figure 1:**
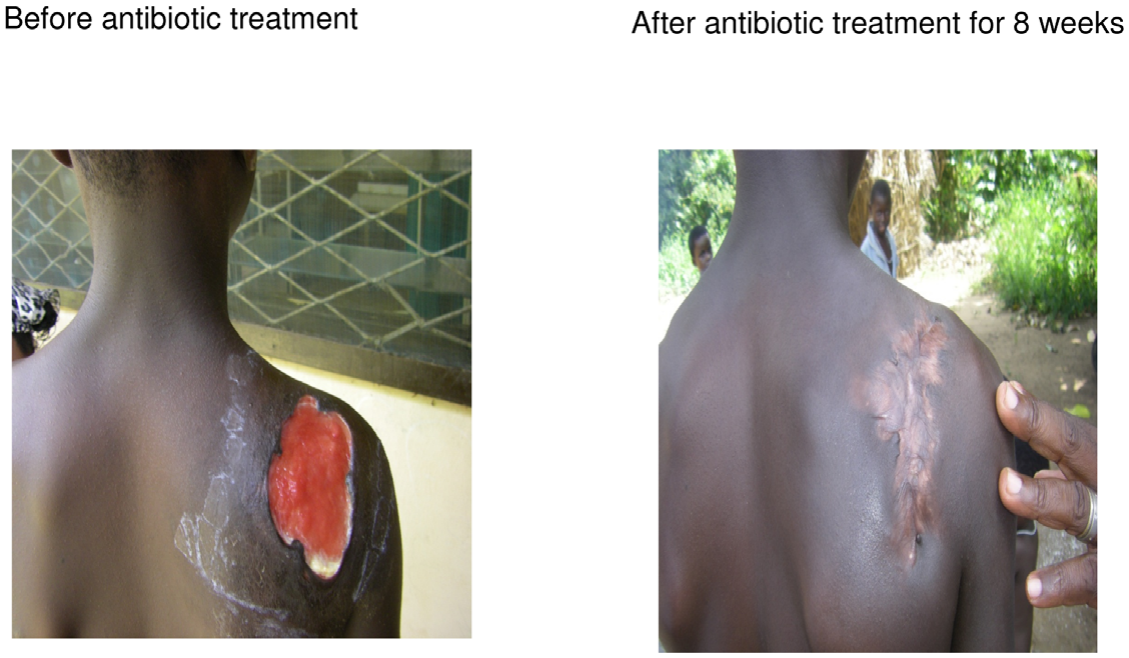
Resolution of Buruli ulcer using antibiotic treatment without surgery.

Before the BUPaT programme, surgery and wound care had been the only available treatment interventions. Improved outcomes of antibiotic therapy have been highly valued by key informants and stakeholders, who regarded it as a breakthrough. Antibiotic treatment has been appreciated because it has minimised recurrence of lesions, which was not possible under the old treatment regime. FGD participants also valued the effect of antibiotic therapy in shrinking lesions and removing necrotic tissue (Figure 1). They made no mention of any negative side-effects of this treatment.

### Challenges of the BUPaT programme

Despite the achievements of the programme, stakeholders and key informants mentioned some major challenges: the inadequacy of ward space to accommodate affected persons who required surgery, the lack of requisite infrastructure in other municipal health centres to perform surgery and the limited health budgets of municipalities. Another challenge was the delay of some affected persons in seeking medical treatment. One stakeholder commented on the challenge of the AH as the main referral and treatment facility for BU as follows:

*We wish we could admit all the patients because some of them report with bad ulcers. However, when there is no space, we can only tell them to go back home and come daily for antibiotic treatment, which does not make economic sense when you live so far away and are poor.*

FGD participants mentioned fears of amputation, loss of livelihoods and the inevitable long absence of the primary care-giver from the home (mostly the mother), when a child is on admission at the hospital, as reasons for delayed treatment. They also expressed concern about feeding (the programme provided two meals a day), transport costs (transport costs of care-givers paying repeated visits to children on admission were not refunded) and difficulties with the continuation of medical treatment if support for feeding and transport was withdrawn. A mother of a treated child explained:

*When you realise that either you or your child has Buruli ulcer and you choose to go to the hospital, you need to prepare financially because of so many reasons: You will have to leave a family behind and you must leave money to take care of them. When you are together as a family it is very easy to cook and share, but when the family is not together it becomes expensive. Now we are given food at the hospital, but what happens to my work when I am away taking care of a sick child; I will lose money. It is easier to seek traditional care and pray it works.*

However, other explanations for delayed medical treatment were linked to misinformation from THs on the likelihood of amputation with medical treatment. Some THs also tried to convince affected persons that herbal treatment was more effective than medical care. Stakeholders indicated that at the beginning of the programme, THs were trained to identify and refer promptly, all cases of BU that were brought to their attention, for appropriate treatment but they acted contrariwise.

FGD participants also expressed difficulties in early diagnosis of their conditions as BU, because of the various presentations of BU infection. For many, it was difficult to know whether cuts, stings, scratches and abrasions were uncomplicated injuries or the beginning of the BU disease. In most cases, these were either unnoticed or dismissed as trivial. As the condition progressed, an assortment of remedies including herbs, balms and hot compresses were applied until BU infection was established; in some cases, after the affected part opened up (revealing the necrotic tissue).

### Stakeholders' suggestions for future services

Stakeholders regarded collaboration, networking and the community-based surveillance system as vital components of the BUPaT programme that had to be sustained. Stakeholders and key informants also mentioned the need to equip existing clinics to serve as treatment centres for wound care and antibiotic treatment. This was considered important to improve access to treatment and reduce severity of reported cases and disabilities, thus reducing the cost burden to the health system.

FGD participants agreed that health education and community-based surveillance activities should continue to increase awareness, improve case detection and encourage early reporting. They also implored the programme to continue to defray transport costs to lessen the economic burden of the disease.

## Discussion

The primary goal of the BUPaT programme was to reduce BU-related suffering and disability through early detection and treatment of cases. Using a mixed method approach, study findings showed the contribution of the health system to BU control in an endemic area in Ghana. Extensive collaboration existed across all levels, (national, municipality and community), which contributed to strengthening the health system. The programme strengthened capacities of health staff in antibiotic treatment and wound care, and trained teachers, MEHOs and CBSVs in health education, screening, early detection and prompt referral for medical treatment. A patient database was also created using recommended WHO forms. WHO-recommended antibiotics improved treatment and cure, particularly for early lesions, thus preventing recurrences. Providing feeding and refund of transport costs proved a useful strategy in encouraging medical care. Irrespective of these achievements, there were still problems of access, accommodation (lack of sufficient ward space), use of traditional treatment, loss to follow-up and non-adherence to treatment.

The broader impact of the BUPaT programme on the health system could be seen in its effects on some of the six building blocks, or subsystems, of the health system, but not on others. With reference to the WHO framework [1], the programme mainly affected governance, human resources, medicines and technology, and health delivery; it had less impact on the financing and information systems. Collaboration and networking among stakeholders strengthened the governance sub-system and improved health delivery of the programme. Training different groups of stakeholders - namely, health staff, CBSVs, MEHOs, teachers and THs - enhanced the human resource sub-system. The administration of WHO-recommended antibiotics improved treatment outcomes and revolutionised the medicines and technologies sub-system. Each of these subsystems contributed to improved health delivery. Minimising expensive surgery by promoting alternative interventions reduced the strain on the limited resources of the finance sub-system. Although the BUPaT programme now routinely compiles patient data using WHO-recommended forms in an electronic database, community epidemiological data are needed for an integrated data system based on community surveillance.

Patient data showed that a significant proportion of admissions comprise children under 15 years-of-age (49 %), consistent with other study findings on the susceptibility of children to BU infection [15], [16]. Even though most cases of BU were not confirmed by laboratory tests, all cases were diagnosed by qualified health staff and surgeons on the basis of WHO clinical case definitions [17]. The BUPaT project aimed to improve early case detection, particularly for nodules, plaques and oedemas, though patient data showed the proportion of patients with pre-ulcer conditions remained less than for ulcer patients. Stakeholders argued that this was not a failure of the programme, however, because people with ulcers who would not previously have used the health system were now seeking medical care instead of remaining with THs.

Consequently, improved awareness has led to treatment of more patients with both pre-ulcerative conditions and ulcers. The reluctance of some people with BU to seek medical care is consistent with findings of other studies [18]–[20]. Studies suggest that the socio-economic impact of BU is a determining factor in the choice of treatment and adherence to medical treatment [6], [7]. Traditional therapy has been the first choice for treatment for some affected persons because of easy local access, compared with the burden of high transport costs, and loss of income due to absence from work while in medical treatment at a distant site [5], [19], [20].

Although increasing community awareness has been bringing more patients to medical treatment, FGDs also showed that various presentations (cuts, bites, stings and abrasions) were not identified as a possible indication of *M. Ulcerans* infection that would benefit from treatment. The effectiveness of antibiotics in preventing recurrences was documented in the patient data. Narratives from stakeholders and key informants referred to this, and they also indicated satisfaction with the minimal cost of antibiotic treatment compared with the high cost of surgery. These findings are consistent with other studies on drug effectiveness [2], [21], [22]. Even though there were no recurrent infections as observed previously when surgery was the only treatment procedure, a significant proportion of patients healed with deformities, most of these patients had ulcers. To minimise deformities, post-operative health care and physiotherapy is required and prosthesis would be needed for amputees. The cost of these services is indeed enormous for an already burdened and poorly resourced rural health service [2], [7]. WHO recommends the need for rehabilitation of patients [23], yet there is paucity of research on its success and integration in the health system.

Based on our study findings, we offer recommendations for effective BU control, particularly for poorly resourced rural health systems. These include health education and community surveillance, collaboration with research laboratories for confirmation of cases, improving access to antibiotic treatment and wound care, integrating BU care with the management of similar diseases and disease mapping:

Our findings show the tremendous impact of health education and community surveillance strategies in BU control. Though this is a laudable community-directed initiative, there is the need for more concerted efforts of the programme to intensify these strategies to reduce BU-related morbidity and increase timely access to medical treatment. All teachers should be trained to identify all forms of *M. ulcerans* infection and refer for medical treatment. School children and others in the community should be encouraged to identify and report suspected cases to teachers, school authorities and community-based surveillance volunteers for verification. Local political commitment is needed by involving chiefs, traditional and religious leaders to support these efforts.

Health education messages should not only focus on creating awareness. They should also emphasise the importance of early reporting and appropriate care to avoid disease sequalae. Messages should encourage affected persons to seek early medical treatment for cuts, abrasions, stings or suspicious swellings. They should correct local ideas about the cause of BU that may discourage appropriate help-seeking. In this regard, it is important that all suspicious pre-ulcerative lesions should be evaluated with laboratory tests. WHO recommends a polymerase chain reaction (PCR) test to confirm cases and diagnosis. Results of this test can be obtained in two days [9]. Given the absence of infrastructure and expertise to perform such analyses, the health system could benefit from collaboration with research laboratories and institutions.

The Ga-West municipality has opened health centres in a few localities to make chemotherapy accessible but these have proven woefully inadequate. There are quite a number of private clinics and maternity homes in both municipalities managed by qualified health personnel who have a large clientele. Integrating them in the health system could boost coverage and access to chemotherapy. The municipal health directorates should assume a supervisory and monitoring role to ensure compliance to case management and chemotherapy protocols.

The cost of managing BU like any other neglected tropical disease is enormous and places a huge strain on a limited rural health budget. Cost-effective interventions should aim at integrating diseases of similar characteristics. Since tuberculosis (TB) case management relies on the Directly Observed Treatment Strategy, all TB centres in the study municipalities could serve as referral treatment centres for identified cases of *M. ulcerans* infection.

Understanding the demographics, epidemiology and geographical distribution of areas that require interventions is critical for cost-effective BU control. The disease is known to be endemic in riverine communities and is attributed to a myriad of factors that include direct exposure to water and swampy areas [24], [25]. These features and documented cases could serve as indices for classifying communities into three categories: priority-endemic areas, requiring the most interventions, endemic and non-endemic, requiring further research to enhance understanding of the disease. First, basic demographic knowledge of all communities must be documented, updated periodically and entered into a central database that will enable mapping and tracking of cases. This is a task for which spatial analytic research is needed.

### Conclusions

Findings demonstrate the role of extensive health education, community-based surveillance, capacity building and collaboration among stakeholders for BU disease control. Treatment with the administration of WHO-recommended antimicrobials has proven effective at least for early lesions. Threats to livelihoods and feeding and transport expenses influence delay to seeking medical care. Findings also indicate the need for an integrated health service delivery approach by incorporating diseases requiring similar antibiotic treatment regimes. A further step towards integration will be to include private health-care providers in the health system to increase access to antibiotic therapy in close proximity to the population. Health education is required in this regard to emphasise the effectiveness of treatment with antibiotics to reduce disease sequalae and the importance of seeking medical treatment for all skin lesions, whether big or small. Evidence from this study suggests that intensifying health education and surveillance would ultimately improve access to treatment for all cases. Further research is needed to explain the role of environmental factors for BU contagion. Health service delivery strategies reported in our study can be adopted for any BU-endemic area in Ghana.
